# Insulin-like growth factor (IGF)-II- mediated fibrosis in pathogenic lung conditions

**DOI:** 10.1371/journal.pone.0225422

**Published:** 2019-11-25

**Authors:** Sara M. Garrett, Eileen Hsu, Justin M. Thomas, Joseph M. Pilewski, Carol Feghali-Bostwick

**Affiliations:** 1 Division of Rheumatology, Department of Medicine, Medical University of South Carolina (MUSC), Charleston, South Carolina, United States of America; 2 Mid Atlantic Permanente Medical Group, Mclean, Virginia, United States of America; 3 Eisenhower Medical Center, Rancho Mirage, California, United States of America; 4 Division of Pulmonary, Allergy, and Critical Care Medicine, University of Pittsburgh, Pittsburgh, Pennsylvania, United States of America; Northumbria University, UNITED KINGDOM

## Abstract

Type 2 insulin-like growth factor (IGF-II) levels are increased in fibrosing lung diseases such as idiopathic pulmonary fibrosis (IPF) and scleroderma/systemic sclerosis-associated pulmonary fibrosis (SSc). Our goal was to investigate the contribution of IGF receptors to IGF-II-mediated fibrosis in these diseases and identify other potential mechanisms key to the fibrotic process. Cognate receptor gene and protein expression were analyzed with qRT-PCR and immunoblot in primary fibroblasts derived from lung tissues of normal donors (NL) and patients with IPF or SSc. Compared to NL, steady-state receptor gene expression was decreased in SSc but not in IPF. IGF-II stimulation differentially decreased receptor mRNA and protein levels in NL, IPF, and SSc fibroblasts. Neutralizing antibody, siRNA, and receptor inhibition targeting endogenous IGF-II and its primary receptors, type 1 IGF receptor (IGF1R), IGF2R, and insulin receptor (IR) resulted in loss of the IGF-II response. IGF-II tipped the TIMP:MMP balance, promoting a fibrotic environment both intracellularly and extracellularly. Differentiation of fibroblasts into myofibroblasts by IGF-II was blocked with a TGFβ1 receptor inhibitor. IGF-II also increased TGFβ2 and TGFβ3 expression, with subsequent activation of canonical SMAD2/3 signaling. Therefore, IGF-II promoted fibrosis through IGF1R, IR, and IGF1R/IR, differentiated fibroblasts into myofibroblasts, decreased protease production and extracellular matrix degradation, and stimulated expression of two TGFβ isoforms, suggesting that IGF-II exerts pro-fibrotic effects via multiple mechanisms.

## Introduction

Chronic fibrosing lung diseases, such as idiopathic pulmonary fibrosis (IPF) and scleroderma/systemic sclerosis (SSc)-associated pulmonary fibrosis, are associated with high rates of mortality and morbidity [1–3]. IPF characteristically affects older males, generally with progressive decline in lung function due to accumulated scarring and fibrosis with a median survival of 2–3 years [2]. Lung disease is the leading cause of death in SSc, which primarily affects women during their child-bearing years and has a median survival of 5–8 years [2]. Though SSc and IPF are distinct diseases, both include fibrosis as a prominent feature [2, 4]. Multiple molecular processes such as oxidative stress, telomere shortening, TGFβ induction, autophagy, myofibroblast activation, and epigenetic, genotypic and phenotypic changes have been implicated in the etiology of these chronic lung fibroses, although the pathogenic mechanism of these diseases remains incompletely understood and only treatments with limited efficacy have been developed [5, 6].

Dysregulation of the insulin-like growth factor (IGF) axis has also been implicated in the pathogenesis of fibrosing lung diseases [7–10]. For example, bronchoalveolar lavage fluid from SSc patients contains increased amounts of IGF-I that can stimulate fibroblast proliferation and collagen deposition [7]. Type 3 and 5 IGF binding proteins (IGFBP) are increased in IPF patient lung and promote extracellular matrix (ECM) deposition [8–11]. Previous work by our laboratory has shown that IGF-II is increased in SSc and signals via the JNK and PI3K pathways [12].

IGF-II is a circulating single chain polypeptide hormone with structural similarity to insulin and IGF-I that is required for normal fetal development [13]. The IGF-II gene is highly imprinted, with primary expression from the paternal allele; loss of imprinting and subsequent biallelic overexpression are mechanistic hallmarks of certain growth disorders [14, 15]. Circulating IGF-II is 5–10 times more prevalent than IGF-I in adults and its dysregulation is also implicated in cardiovascular disease, diabetes mellitus, metabolic syndrome, obesity, and myriad oncologies [14–19]. Free IGF-II primarily binds to three receptors: its cognate IGF-II receptor (IGF2R, homologous to the mannose-6-phosphate receptor), the IGF-I receptor (IGF1R, CD221, or JTK13), and the insulin receptor (IR, INSR, CD220, or HHF5), albeit with respectively decreasing affinities. The IGF1R and IR are tetrameric tyrosine kinase glycoprotein receptors; thus, upon ligand binding, they undergo auto-phosphorylation at several sites along the cytoplasmic tail and activate docking proteins that signal downstream mediators for signal transduction [20]. While IGF1R, IGF2R, and IR all have extracellular, transmembrane, and cytoplasmic portions, the cytoplasmic tail of the single-pass transmembrane IGF2R is relatively short, suggesting that its ability to signal intracellularly may be limited compared to the other two receptors and that its binding of IGF-II may serve to limit excess levels of bioavailable IGF-II in the circulation by mediating its endocytosis and lysosomal degradation [13, 15, 19, 21]. The membrane-bound IGF2R can be cleaved by proteases to release a soluble IGF2R containing only the extracellular portion of the receptor, though its specific role has not been delineated [21].

Therefore, the purpose of this work was to study the mechanism of IGF-II-mediated fibrosis, including the role of its receptors and the effect of IGF-II overexpression on ECM production, secretion, and deposition. Herein we show that IGF-II signals primarily through IGF1R and IGF1R/IR to promote a pro-fibrotic environment by down-regulation of matrix metalloproteinase (MMP) 3, upregulation of TGFβ isoforms 2 and 3, and secretion of tissue inhibitor of metalloproteinase (TIMP) 1 and TIMP4.

## Materials and methods

### Cell culture

Primary lung fibroblasts isolated from normal lung tissue of organ donors whose lungs were not used in transplant surgery and explanted lungs of patients with IPF or SSc who underwent lung transplantation at the University of Pittsburgh Medical Center were cultured as previously described under a protocol approved by the University of Pittsburgh Institutional Review Board [8]. Age distributions for donors used in this study are shown in S1 Fig. Fibroblasts were maintained in DMEM supplemented with 10% FBS and 1% antibiotic/antimycotic at 37°C/ 5% CO_2_ under humidifying conditions and utilized between passages two and seven, inclusively.

### Reagents and antibodies

DMEM for cell culture was purchased from Corning (Corning, NY, USA). Fetal bovine serum, protease inhibitor cocktail, and αSMA antibody were from Sigma-Aldrich (St. Louis, MO, USA). IGF1R inhibitor I-OMe-Tyrphostin AG 538 was from Calbiochem (San Diego, CA, USA). Recombinant human IGF-II and anti-IGF-II antibody were purchased from R&D Systems (Minneapolis, MN, USA). Collagen, Fibronectin, and GAPDH antibodies were from Santa Cruz (Santa Cruz, CA, USA). IGF1R, IGF2R, IR-β, phospho-SMAD and total SMAD antibodies were purchased from Cell Signaling Technology (Danvers, MA, USA). Lipofectamine^®^ 2000 was purchased from Invitrogen (Carlsbad, CA, USA). IGF1R, IR, and isotype control antibodies used in neutralization experiments were obtained from GroPep Bioreagents (Thebarton SA 5031, Australia). IGF1R, IGF1R, IR, and scrambled siRNA were purchased from Thermo Fisher Scientific (Waltham, MA, USA). HRP-conjugated anti-mouse IgG was from Promega (Madison, WI, USA) and anti-rabbit-HRP IgG was from GE Healthcare Life Sciences (Chicago, IL, USA).

### RNA isolation, reverse transcription, and qPCR

RNA was isolated from fibroblasts using the TRIzol^™^ separation method per manufacturer’s instructions (Invitrogen, Carlsbad, CA, USA) and resuspended in purified water. RNA was quantified using a NanoDrop Lite spectrophotometer (Thermo Fisher Scientific, Waltham, MA, USA). cDNA was prepared from 1 μg RNA per 20 μL cDNA reaction using oligo (dT) and SuperScript^®^ IV First-Strand Synthesis system (Thermo Fisher) on a C1000 Touch^™^ Thermal Cycler (Bio-Rad Laboratories, Hercules, CA, USA). PCR was performed using 1 μL cDNA in a 10 μL reaction including best-coverage TaqMan^®^ Gene Expression primers on an Applied Biosystems StepOne Real-Time PCR System (Thermo Fisher). Primers used include *ACTA2* (Hs00426835_g1), *B2M* (Hs00187842_m1), *Collagen 1A1* (Hs00164004_m1), *Fibronectin 1* (Hs00365052_m1), *GAPDH* (H202758991_g1), *IGF1R* (Hs00609566_m1), *IGF2R* (Hs00974474_m1), *IR* (Hs00961560_m1), *MMP3* (Hs00968305_m1), *TGFβ1* (Hs00998133_m1), *TGFβ2* (Hs00234244_m1), *TGFβ3* (Hs01086000_m1), *TIMP1* (Hs00171558_m1), and *TIMP4* (Hs00162784_m1). Gene expression was determined by the delta-delta Ct method [22].

### Immunoblotting

Fibroblasts were serum-starved overnight prior to stimulation with IGF-II (200 ng/mL) or vehicle (PBS). Equal amounts of proteins from cellular lysates or supernatants resolved in SDS-PAGE gels were subsequently transferred to nitrocellulose or PVDF membranes. Membranes were blocked, incubated with primary antibody, washed in TBS-Tween, incubated with peroxidase-conjugated secondary antibody, washed in TBS-tween, then proteins were visualized using enhanced chemiluminescence (ECL Western Blot substrate, PerkinElmer, Waltham, MA) on a FluorChem R System (ProteinSimple, San Jose, CA, USA) or autoradiography. Densitometry was performed with ImageJ or AlphaView software.

### Neutralization, inhibition, and knockdown

For neutralization of IGF-II, fibroblasts were serum-starved overnight, then treated with neutralizing antibody for 1 hr prior to stimulation with IGF-II (200 ng/mL; this concentration was used inclusively for all stimulation experiments). Similarly, for inhibition of IGF1R with the specific tyrosine kinase inhibitor I-OMe-Tyrphostin AG 538, fibroblasts were serum-starved overnight, treated with 5–20 μM inhibitor for 1 hr, then stimulated with IGF-II. In silencing experiments, cells 80–90% confluent were transfected with 100 nM of siRNA (directed against IGF1R, IGF2R, or IR) or scrambled control with Lipofectamine^®^ 2000 for 24 hr per manufacturer’s instructions. Fibroblasts were then serum-starved and stimulated with IGF-II for 24–48 hr.

### ELISA

Protein secretion was measured in fibroblast supernatants using commercially available sandwich ELISA kits (MMP3: Sigma-Aldrich, St. Louis, MO, USA; TGFβ1: R&D Systems, Minneapolis, MN; TIMP1: Invitrogen, Camarillo, CA, USA; TIMP4: Thermo Fisher Scientific, Waltham, MA, USA;) per manufacturer’s instructions. Supernatants were assayed undiluted except in the TIMP1 assay (diluted 1:50 with Standard Diluent Buffer).

### Reverse gelatin zymography

Equal volumes of supernatants in non-reducing sample buffer were loaded into 10% acrylamide gels containing 2.5 mg gelatin and 1/5 vol/vol TGFβ1-conditioned media in the resolving portion of the gel. Resolved gels were washed (0.25% Triton X-100 in 50 mM Tris, pH 7.6) then incubated (10 mM CaCl_2_, 1% Triton X-100, 0.2% sodium azide in 50 mM Tris, pH 7.6) overnight at 37°C. Gels were stained with coomassie blue, then destained (10% acetic acid, 40% methanol, 50% water) and imaged on a FluorChem R System (ProteinSimple, San Jose, CA, USA). Densitometry was performed with AlphaView software.

### Statistics

Mean values and standard errors of the mean of all treatments were analyzed for statistical significance of p < 0.05 utilizing Student’s t test or analysis of variance with an appropriate post-hoc test.

## Results

### Steady-state and IGF-II-stimulated changes in receptor gene expression

Our previous study has shown that IGF-II transcript and protein levels are significantly increased in lung fibroblasts of patients with SSc compared to normal lung (NL) fibroblasts [12]. IGF-II has been shown to increase mRNA and protein levels of extracellular proteins, including increasing fibronectin in IPF fibroblasts [23] and amplifying collagen and fibronectin in NL and SSc fibroblasts [12]. We expanded these findings by showing that IGF-II regulates ECM production at the transcriptional level and identified a significant upregulation of *collagen* and *fibronectin* transcripts in IPF and SSc fibroblasts (S1 Fig).

**Fig 1:**
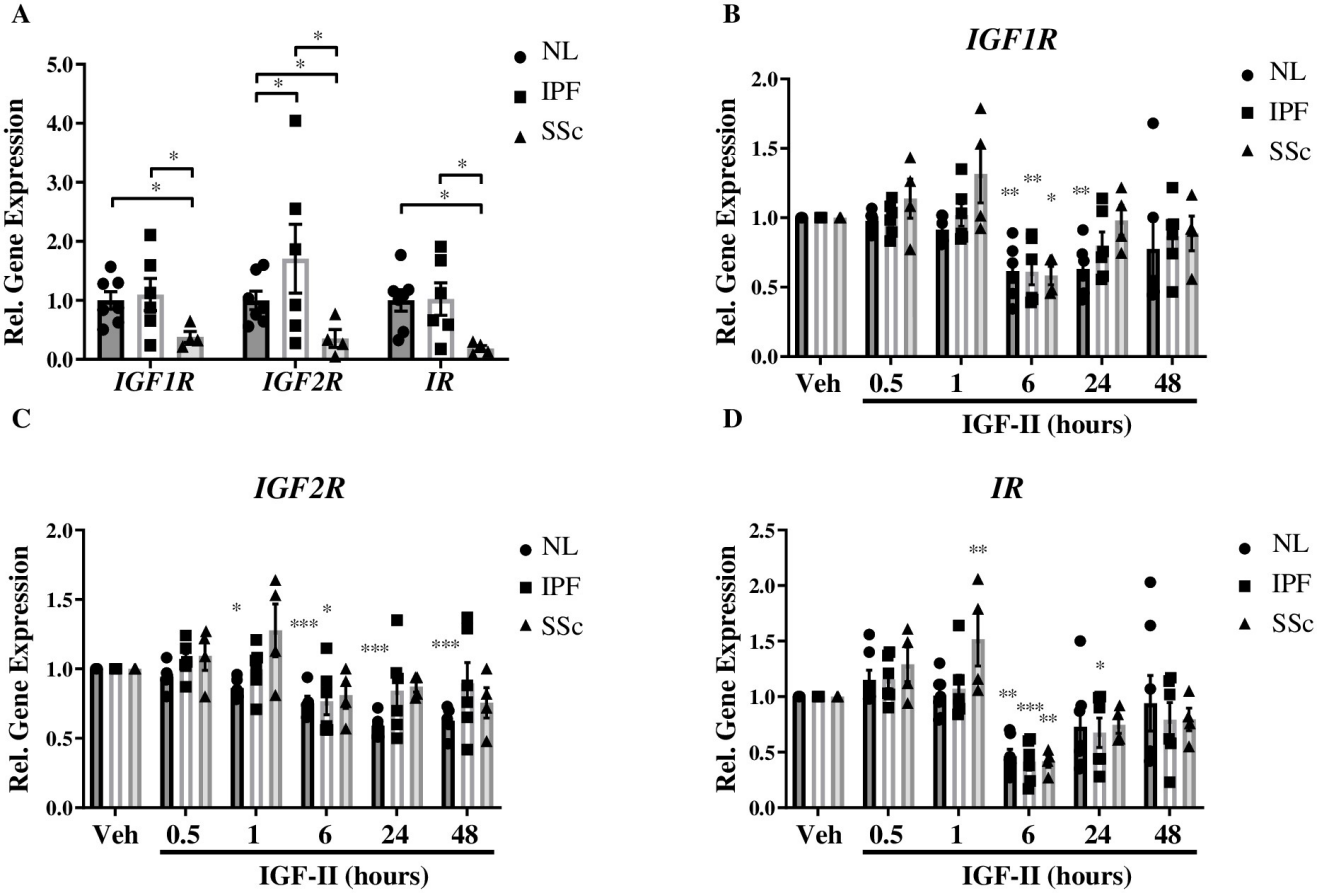
Steady-state and IGF-II-stimulated changes in receptor gene expression. Basal (A) and IGF-II (200 ng/mL)-mediated gene expression of *IGF1R* (B), *IGF2R* (C), and *IR* (D) receptors in Normal Lung (NL), IPF, and SSc fibroblasts normalized to *GAPDH* housekeeping gene. N = 3–6. *p<0.05, **p<0.01, ***p<0.001 by paired 2-tailed Student’s T Test (A) and 1-way ANOVA with Dunnett’s multiple comparison test compared to vehicle (PBS, B-D).

We next sought to determine the mechanism of IGF-II-induced fibrosis, beginning with the role of the IGF-II-responsive receptors, IGF1R, IGF2R, and IR. Basal gene expression levels of IGF-II receptors in unstimulated fibroblasts were assessed to determine if any differences exist in receptor steady-state mRNA levels in NL, IPF, and SSc fibroblasts (Fig 1A). Steady-state gene expression levels of *IGF2R* were higher in IPF compared to NL and SSc fibroblasts. All three baseline receptor levels were significantly lower in SSc compared to NL and IPF fibroblasts.

To determine if there were major differences in receptor transcript response to ligand stimulation, NL, IPF, and SSc fibroblasts were treated with IGF-II, and receptor mRNA levels were analyzed at different time points (Fig 1B–1D). IGF-II stimulation in NL significantly decreased *IGF1R* transcript from 6–24 hr, after which the expression level returned to baseline (Fig 1B). *IGF1R* gene expression was only decreased at 6 hr post IGF-II treatment in both IPF and SSc, with no other significant transcript variations. IGF-II led to a significant decrease in *IGF2R* mRNA in NL from 1–48 hr and in IPF at 6 hr, but did not cause any significant changes in SSc (Fig 1C). *IR* transcripts in all fibroblasts were significantly decreased at 6 hr by IGF-II, while additionally decreased in IPF at 24 h and increased in SSc at 1 hr (Fig 1D).

Thus, in general, IGF-II stimulation significantly down-regulated gene expression of the receptors tested in all of the fibroblast strains at 6 hr, with a return to baseline level by 48 hr of stimulation, the only exception being an early and persistent reduction in *IGF2R* in NL fibroblasts that did not recover to baseline levels over time. Hence in the context of IGF2R expression, IPF and SSc reacted similarly to IGF-II, but differently from NL. The downregulation of *IGF1R* and *IR* transcripts at 6 hr in response to IGF-II stimulation in all fibroblast lines likely represents normal ligand-induced receptor desensitization and down-regulation. The lack of more than a transient *IGF2R* downregulation in IPF and SSc may represent a mechanism for IGF-II-mediated fibrosis.

### Steady-state and IGF-II-stimulated changes in receptor protein expression

**Fig 2:**
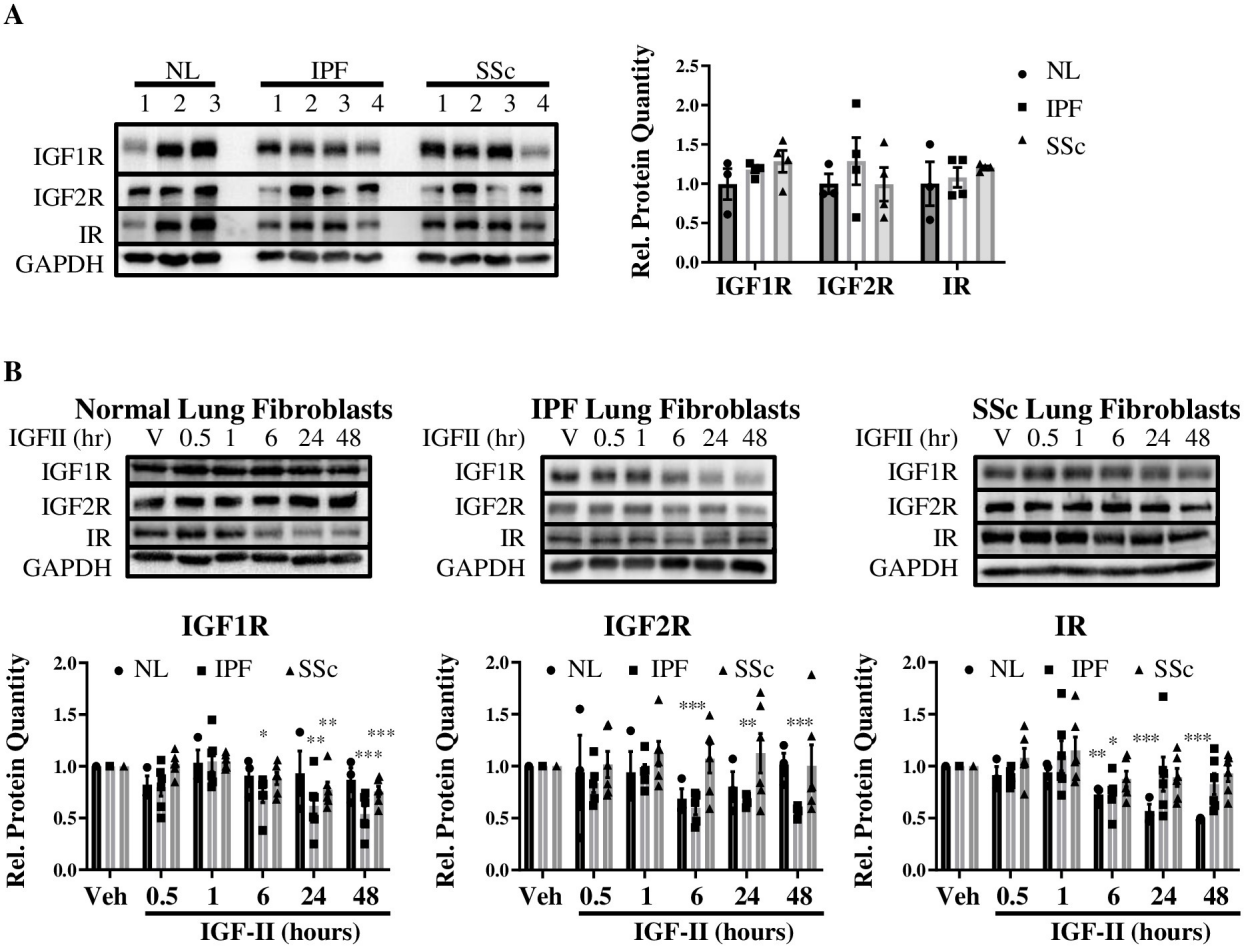
Steady-state and IGF-II-stimulated changes in receptor protein expression. Basal (A) and IGF-II (200 ng/mL)-stimulated protein expression of IGF1R, IGF2R, and IR receptors (B) in Normal Lung (NL), IPF, and SSc fibroblasts normalized to GAPDH housekeeping protein. Quantification of protein expression was graphed as histograms with mean +/- standard error of the mean and representative immunoblots included. N = 3–6. *p<0.05, **p<0.01, ***p<0.001 by paired 2-tailed Student’s T Test (A) and 1-way ANOVA with Dunnett’s multiple comparison test compared to vehicle (PBS, B).

Despite steady-state differences in receptor mRNA levels between NL, IPF, and SSc, the unstimulated basal protein levels of these receptors were not significantly different between fibroblast populations (Fig 2A). Whereas IGF1R protein was unaltered by IGF-II-stimulation in NL, IGF1R was significantly decreased in both IPF (6–48 hr) and SSc (24–48 hr) with stimulation (Fig 2B). IGF-II caused a persistent decrease in IGF2R in IPF (6–48 hr) with no significant changes in NL or SSc. IR protein was decreased upon IGF-II stimulation in NL (6–48 hr) and IPF (6 hr), but unchanged in SSc. Stimulus-driven down-regulation of IR in NL fibroblasts may ultimately serve as a mechanism of reducing the available pool of receptors capable of being activated, thus acting as a dampening mechanism for receptor activation and subsequent signal transduction in NL, the likes of which is transient (in IPF at 6 hr) or absent (in SSc) in fibrosing lung conditions.

### Blockade of endogenous IGF-II or its receptors abrogates ECM component production

**Fig 3:**
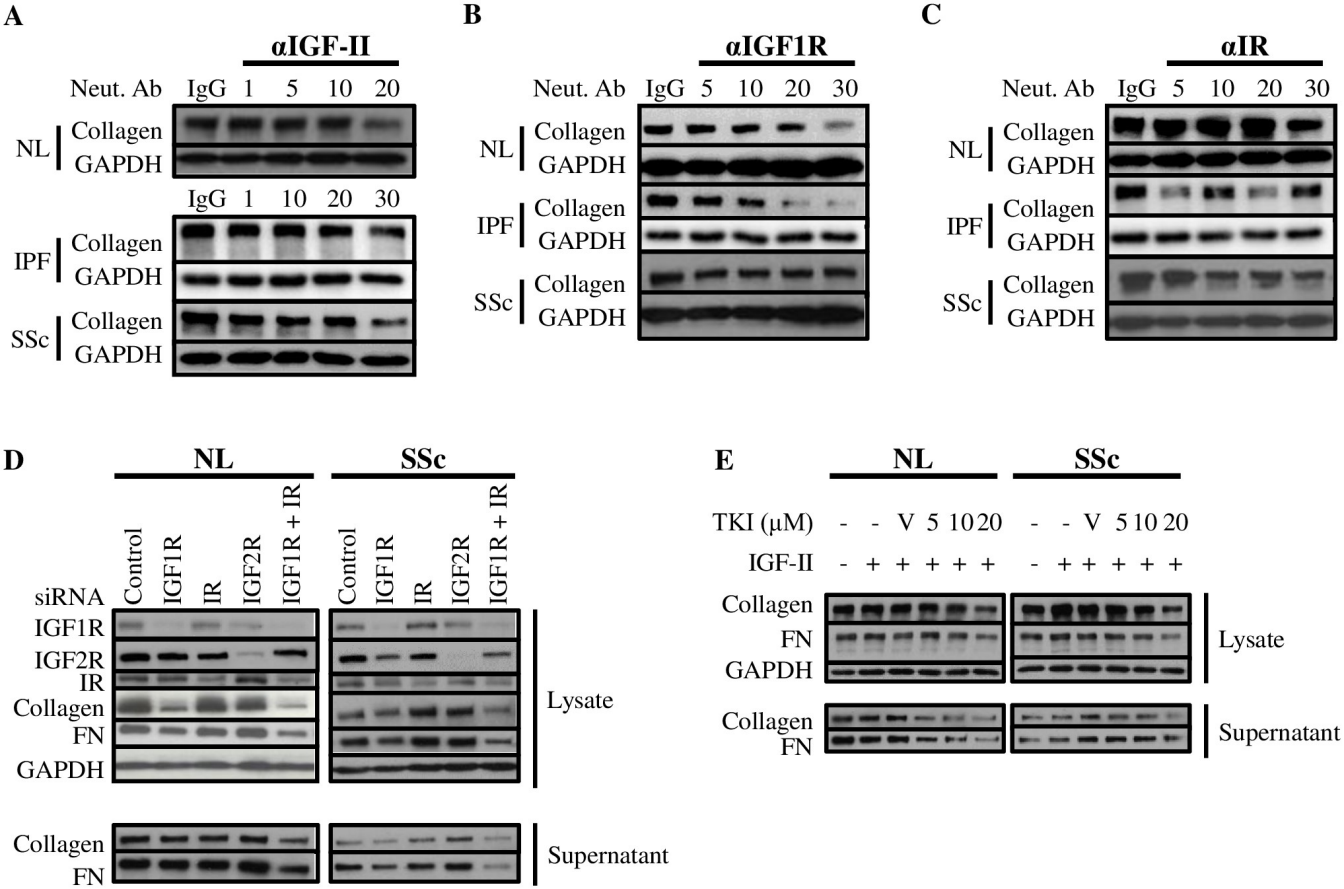
Blockade of endogenous IGF-II or its receptors abrogates ECM component production. NL, IPF, and SSc lung fibroblasts were treated with neutralizing antibodies to endogenous IGF-II (A), IGF1R (B), IR (C), or appropriate isotype control (respective highest antibody concentration, 20–30 μg/mL) for 1 hr, followed by stimulation with IGF-II (200 ng/mL) for 48 hr and lysates were probed for Collagen and GAPDH by immunoblot. D: Lung fibroblasts were subjected to knockdown of IGF1R, IR, IGF2R, dual knockdown of IGF1R + IR, or scrambled siRNA in the presence of IGF-II (200 ng/mL) and probed for protein expression of Collagen, Fibronectin (FN), and GAPDH in lysates and supernatants. E: Lung fibroblasts were treated with increasing concentrations of IGF1R tyrosine kinase inhibitor (TKI, Tyrphostin AG 538) in the presence of 200 ng/mL IGF-II and probed for protein expression of Collagen, Fibronectin, and GAPDH in lysates and supernatants. Representative immunoblots from at least three experiments.

To further investigate the contribution of IGF-II, IGF1R, and IR to the fibrotic process, fibroblasts were treated with neutralizing antibody or isotype control for one hour prior to exogenous IGF-II stimulation for 48 hr and analyzed for changes in the abundant ECM protein collagen type I (Fig 3A–3C). Neutralization of endogenous IGF-II decreased collagen levels at the highest dose of antibody in NL (20 μg/mL), IPF (30 μg/mL), and SSc (30 μg/mL) fibroblasts (Fig 3A). Neutralization of IGF1R led to a decrease in collagen production in NL (30 μg/mL) and IPF (20–30 μg/mL), with minimal changes to SSc collagen levels (Fig 3B). IR neutralization resulted in a decrease in NL fibroblast collagen (30 μg/mL), a variable decrease in IPF fibroblast collagen (1–30 μg/mL), and a decrease in SSc fibroblast collagen (5–30 μg/mL; Fig 3C). Therefore, endogenous IGF-II, IGF1R, and IR each contribute to the IGF-II-mediated fibrotic signal, as represented through diminished collagen production by NL, IPF, and SSc fibroblasts.

### Disruption of IGF1R and IGF1R/IR blocks ECM production and deposition

To examine the contribution of cognate IGF-II receptors to IGF-II-mediated fibrosis, siRNA knockdown and pharmacological inhibition were utilized in NL and SSc fibroblasts, since SSc fibroblasts showed a more pronounced induction of *fibronectin* and *collagen* expression with IGF-II stimulation than IPF fibroblasts (S1 Fig). First, IGF1R, IR, and IGF2R were subjected to siRNA knockdown either individually or in combination (IGF1R + IR) with targeted siRNA (Fig 3D). IGF1R siRNA in NL significantly decreased intracellular collagen, modestly decreased fibronectin, and minimally decreased secreted collagen and fibronectin. Knockdown of IGF1R in SSc reduced lysate and supernatant fibronectin with minimal effect on collagen in either fraction. In contrast, knockdown of IR or IGF2R did not significantly decrease collagen or fibronectin in lysate or supernatant in NL or SSc. However, dual knockdown of IGF1R + IR significantly and drastically decreased both collagen and fibronectin in lysates and supernatants in both NL and SSc.

Tyrphostin AG 538, a specific inhibitor of the IGF1R tyrosine kinase (TKI), was used to confirm the contribution of IGF1R in IGF-II-stimulated fibrosis in NL and SSc (Fig 3E). In both NL and SSc, intracellular collagen and fibronectin were reduced in a dose-dependent manner in response to IGF1R TKI (10 μM– 20 μM). In NL supernatant, collagen and fibronectin were significantly reduced with as low as 5 μM IGF1R TKI. Collagen in SSc supernatant was also similarly decreased; however, SSc supernatant fibronectin levels were reduced only with the highest concentration of inhibitor (20 μM).

Based on results from the neutralizing antibody, siRNA knockdown, and pharmacological inhibition experiments, we conclude that IGF1R represents a specific and prominent contributor to IGF-II-mediated ECM production and deposition. Combining the fact that IGF1R and IR can form a hybrid disulfide-linked heterodimer and that dual knockdown of IGF1R + IR more effectively reduced collagen and fibronectin than IGF1R or IR alone, the hybrid receptor most likely represents a significant receptor species contributing to fibrosis mediated by IGF-II, and thus also represents a likely therapeutic target for the treatment of SSc.

### IGF-II promotes an intracellular and an extracellular fibrotic environment

**Fig 4:**
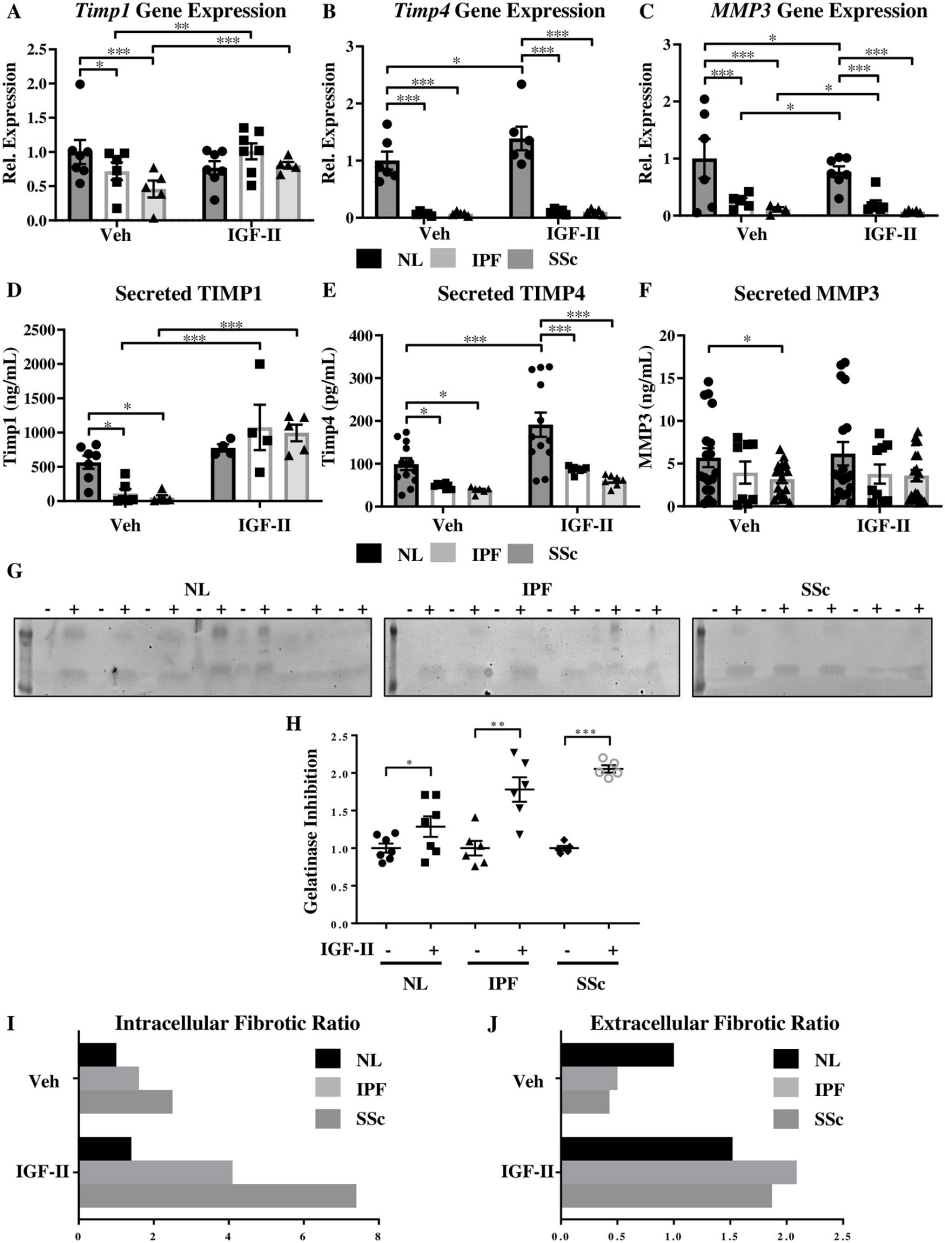
IGF-II promotes an intracellular and an extracellular fibrotic environment. Transcript levels of *TIMP1* (A), *TIMP4* (B), and *MMP3* (C) normalized to *GAPDH* housekeeping gene by qRT-PCR and secreted TIMP1 (D), TIMP4 (E), and MMP3 (F) levels in supernatant by ELISA in NL, IPF, and SSc fibroblasts treated with vehicle (PBS) or IGF-II (200 ng/mL) for 48 hr. N = 5–10. *p<0.05, **p<0.01, ***p<0.001 by 1-way ANOVA with Dunnett’s multiple comparison test. G: Reverse gelatin zymography of supernatants from fibroblasts treated with vehicle (-) or IGF-II (200 ng/mL, +) for 48 hr and quantification of relative gelatinase inhibition (H). Individual samples are represented with mean +/- standard error of the mean as indicated. N = 5–7. *p<0.05, **p<0.01, and ***p<0.001 by paired 2-tailed Student’s T test. Fibrotic ratios of relative *TIMP1* & *TIMP4* to relative *MMP3* transcript (I, intracellular) and secreted protein (J, extracellular) levels in NL, IPF, and SSc fibroblasts. Ratio was calculated by taking the average of relative *TIMP1* and relative *TIMP4*, then dividing by relative *MMP3*. High fibrotic ratio indicates increased ECM deposition and/or decreased ECM breakdown.

Extracellular matrix breakdown is mediated in part by the matrix metalloproteinase (MMP) family of enzymes, while ECM deposition can be promoted by MMP inhibition via the tissue inhibitor of matrix metalloproteinases (TIMPs). Impairment of this protease system has long been speculated in pulmonary fibroses [24, 25]. The relative contribution of representative members of each of these families of enzymes on ECM was evaluated in fibroblasts in the presence and absence of IGF-II stimulation (Fig 4). Steady-state and IGF-II-mediated changes in intracellular transcript levels and secretion of TIMP1, TIMP4, and MMP3 were analyzed in NL, IPF, and SSc fibroblasts by real-time PCR (Fig 4A–4C) and ELISA (Fig 4D–4F). *TIMP1* mRNA levels in unstimulated IPF and SSc fibroblasts were lower than in NL fibroblasts under basal conditions (28% lower in IPF and 54% lower in SSc; Fig 4A). IGF-II stimulation significantly increased *TIMP1* in IPF (40% increase) and SSc (76% increase), while non-significantly decreasing *TIMP1* in NL. Basal *TIMP4* transcript levels were significantly higher in NL fibroblasts compared to fibroblasts from patients: IPF and SSc fibroblasts contained approximately 10% of the *TIMP4* transcript level of NL (Fig 4B). The addition of IGF-II significantly increased *TIMP4* gene expression in NL fibroblasts but not in IPF or SSc. *MMP3* transcripts were significantly lower in IPF (75% less) and SSc (90% less) compared to NL; addition of IGF-II significantly decreased *MMP3* transcript levels in all populations (20% reduction in *MMP3* mRNA in NL, a 45% decrease in IPF, and a 43% decline in SSc; Fig 4C).

Secreted TIMP1, TIMP4, and MMP3 levels were also quantified by ELISA in supernatants of NL, IPF, and SSc fibroblasts treated with vehicle (PBS) or IGF-II (200 ng/mL) for 48 hr (Fig 4D–4F). The amount of TIMP1 secreted by unstimulated NL exceeded that by either IPF (20% of NL) or SSc (9% of NL; Fig 4D). Following IGF-II stimulation, secreted TIMP1 was non-significantly elevated in NL, but drastically increased in IPF (10-fold) and SSc (20-fold) fibroblasts compared to baseline levels, suggesting that gene expression and protein secretion for TIMP1 followed similar trends. Steady-state secreted TIMP4 levels in IPF and SSc were significantly lower than in NL, with TIMP4 levels approximately two-times higher in NL (Fig 4E). In the presence of IGF-II, TIMP4 secretion was significantly increased in NL, but not in IPF or SSc; overall, gene expression and protein secretion trends aligned well for TIMP4. The steady-state level of secreted MMP3 was similar between NL and IPF, but significantly lower in SSc compared to NL (Fig 4F). The addition of IGF-II did not significantly change the secretion of MMP3 in any of the fibroblast populations.

The two human gelatinolytic proteinases, MMP2 (gelatinase A) and MMP9 (gelatinase B), can be inhibited by TIMPs [26]. We examined the effects of IGF-II on gelatinase activity using reverse gelatin zymography in 48 hr-treated supernatants from NL, IPF, and SSc fibroblasts (Fig 4G and 4H). The addition of IGF-II inhibited gelatinase in NL fibroblasts; the effect was more pronounced in IPF, and even more prominent in SSc.

To get a better indication of the pro-fibrotic state of the intracellular environment and of the ECM milieu, ratios of TIMPs:MMPs based on transcript (Fig 4I) and protein (Fig 4J) levels were determined. The ratio of TIMP1 + TIMP4 to MMP3 was calculated as the ‘fibrotic ratio.’ Under steady-state conditions, the intracellular fibrotic ratio was 60% higher in IPF fibroblasts and 150% higher in SSc fibroblasts compared to NL fibroblasts (Fig 4I). Fibrotic ratios were skewed even further in the presence of IGF-II: there was a 40% increase in NL compared to unstimulated NL, >150% increase in IPF, and ~200% increase in SSc compared to respective vehicle-treated fibroblasts. IGF-II additionally promoted a fibrotic environment extracellularly, with a 52% increase in NL and >300% increase in IPF and SSc upon IGF-II stimulation (Fig 4J). Addition of IGF-II promotes a profibrotic environment and further exacerbates the profibrotic state of IPF and SSc both intracellularly and extracellularly, significantly and drastically altering the fibrotic milieu, thus favoring an environment of ECM accumulation and/or reduced ECM degradation.

### IGF-II stimulates TGFβ isoform expression and signaling

**Fig 5:**
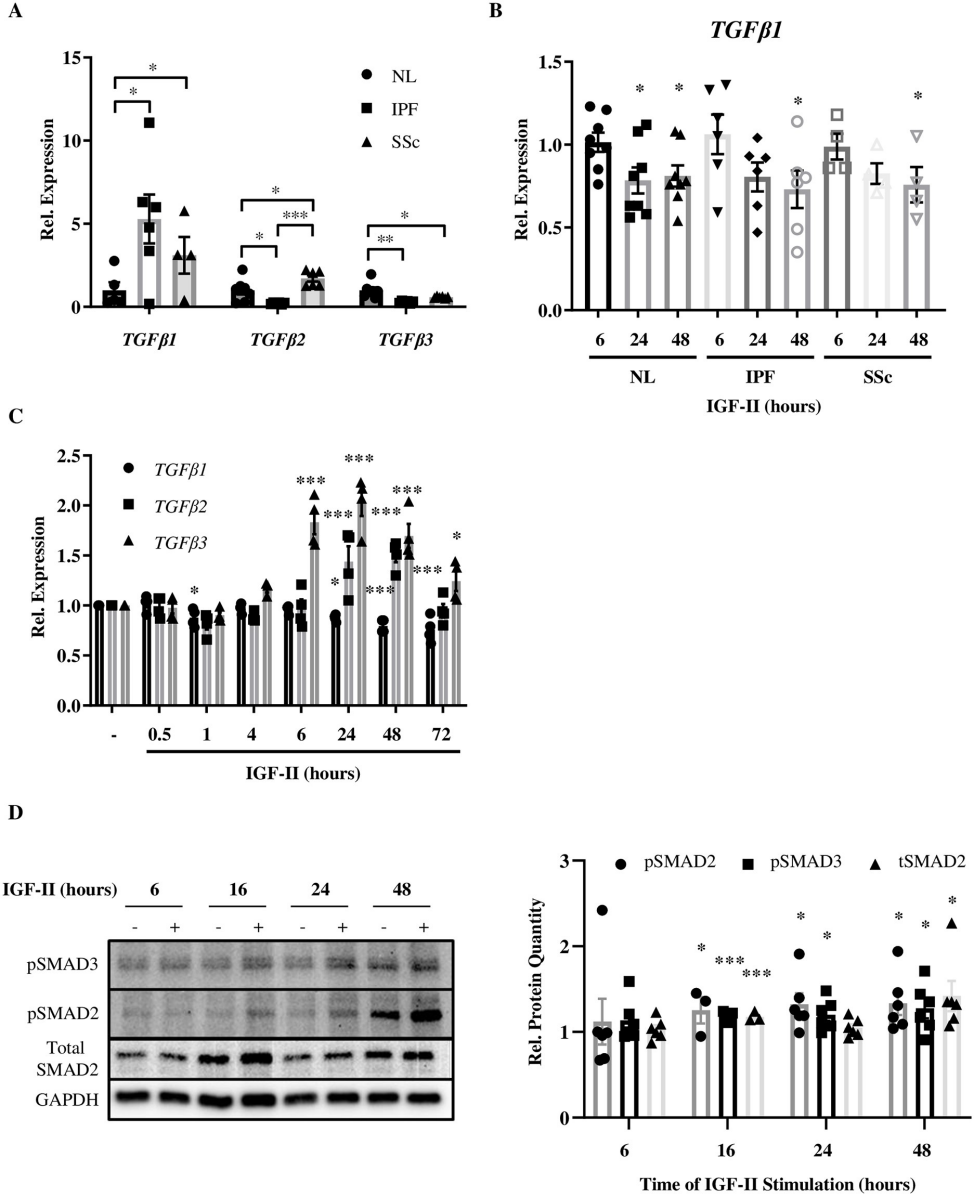
IGF-II stimulates TGFβ isoform expression. A: Steady-state expression of *TGFB1*, *TGFB2*, and *TGFB3* in NL, IPF, and SSc fibroblasts. B: *TGFB1* gene expression in NL, IPF, and SSc fibroblasts over time with IGF-II stimulation (200 ng/mL). C: Gene expression of *TGFB1*, *TGFB2*, and *TGFB3* in NL fibroblasts treated with IGF-II (200 ng/mL) or vehicle (PBS). D: Representative images and quantification of SMAD2/3 activation in NL fibroblasts over time. N = 3–8. Histograms show data normalized to GAPDH presented as mean +/- standard error of the mean with significance by 1-way ANOVA with Dunnett’s multiple comparison test indicated as *p<0.05, **p<0.01, and ***p<0.001. (-): vehicle (PBS), (+): IGF-II (200 ng/mL).

As TGFβ1 is the typical growth factor used to induce fibrosis *in vitro* [5], we sought to determine if IGF-II regulates TGFβ1 expression *in vitro*. Levels of TGFβ1 have been reported to be higher in patients with lung pathologies including IPF and SSc and we have confirmed this finding (Fig 5A, S1 Fig) [27–29]. Whereas steady-state levels of *TGFβ2* and *TGFβ3* were consistently lower in IPF fibroblasts, *TGFβ2* was higher and *TGFβ3* was lower in SSc fibroblasts compared to NL fibroblasts (Fig 5A). Despite higher steady-state levels of *TGFβ1* gene expression in IPF and SSc compared to NL fibroblasts, IGF-II stimulation led to modestly decreased *TGFβ1* transcript levels over time in these fibroblast populations (Fig 5B).

Since *TGFβ1* was decreased by IGF-II in all three fibroblast strains, we compared its levels to those of the other isoforms, TGFβ 2 & 3, in response to IGF-II in NL fibroblasts (Fig 5C). *TGFβ1* transcripts were significantly decreased by IGF-II (0.5 hr, 24–72 hr), while both *TGFβ2* (24–48 hr) and *TGFβ3* (6–72 hr) were significantly increased with IGF-II treatment. To characterize the mechanistic signaling events surrounding the upregulation of TGFβ2/3, NL lysates were probed for activation of canonical SMAD2/3 (Fig 5D). Relative SMAD2 and SMAD3 phosphorylation induced by IGF-II was significantly increased from 16–48 hr stimulation and total SMAD2 was increased at 16 hr and 48 hr.

### IGF-II contributes to transdifferentiation of NL fibroblasts into myofibroblasts

**Fig 6:**
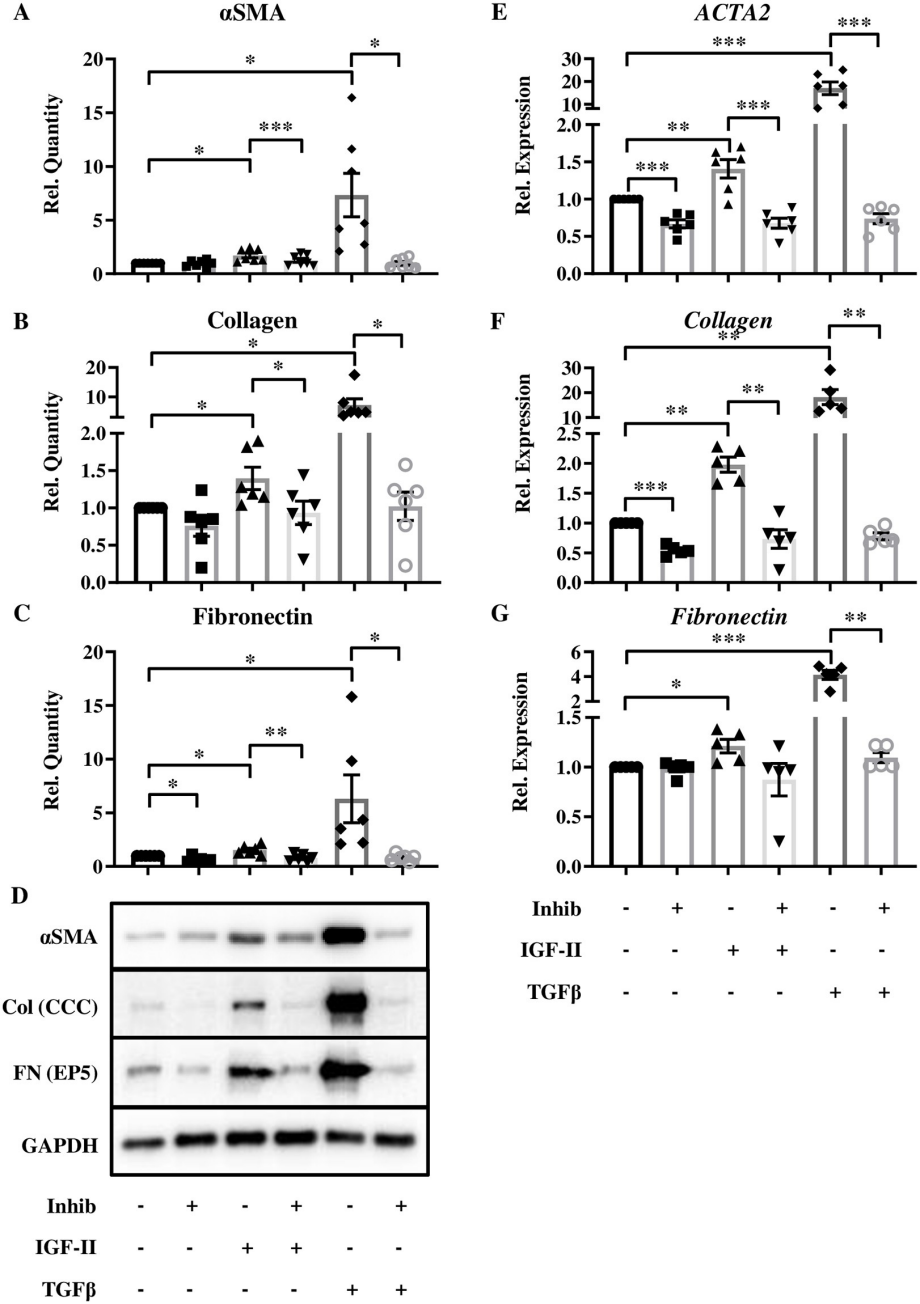
IGF-II contributes to transdifferentiation of NL fibroblasts into myofibroblasts. Expression of αSMA (A), Collagen (B), and Fibronectin (C) proteins with representative images (D) and *αSMA/ACTA2* (E), *Collagen* (F), and *Fibronectin* (G) transcripts in NL after IGF-II (200 ng/mL) stimulation for 96 hr (protein) or 72 hr (transcript) following 1 hr pre-treatment with TGFBR1 inhibitor (SB431542). N = 6–7. Histograms are mean +/- standard error of the mean with significance by 1-way ANOVA with Dunnett’s multiple comparison test indicated as *p<0.05, **p<0.01, and ***p<0.001.

Abundant secretion of ECM proteins and expression of alpha-smooth muscle actin (αSMA) are markers of myofibroblasts. These cells can differentiate from multiple cell types, including fibroblasts, by treatment with a variety of factors, such as TGFβ1 [5, 30]. To determine if IGF-II can mediate a fibroblast-to-myofibroblast conversion, we assayed for the production of αSMA and compared it to TGFβ1-mediated regulation of ECM components collagen and fibronectin (Fig 6). IGF-II treatment led to a significant upregulation of the myofibroblast marker αSMA (Fig 6A and 6D), as well as the ECM proteins collagen (Fig 6B and 6D), and fibronectin (Fig 6C and 6D). Transcript levels of *αSMA*/*ACTA2* (Fig 6E), *collagen* (Fig 6F), and *fibronectin* (Fig 6G) followed similar trends.

**Fig 7:**
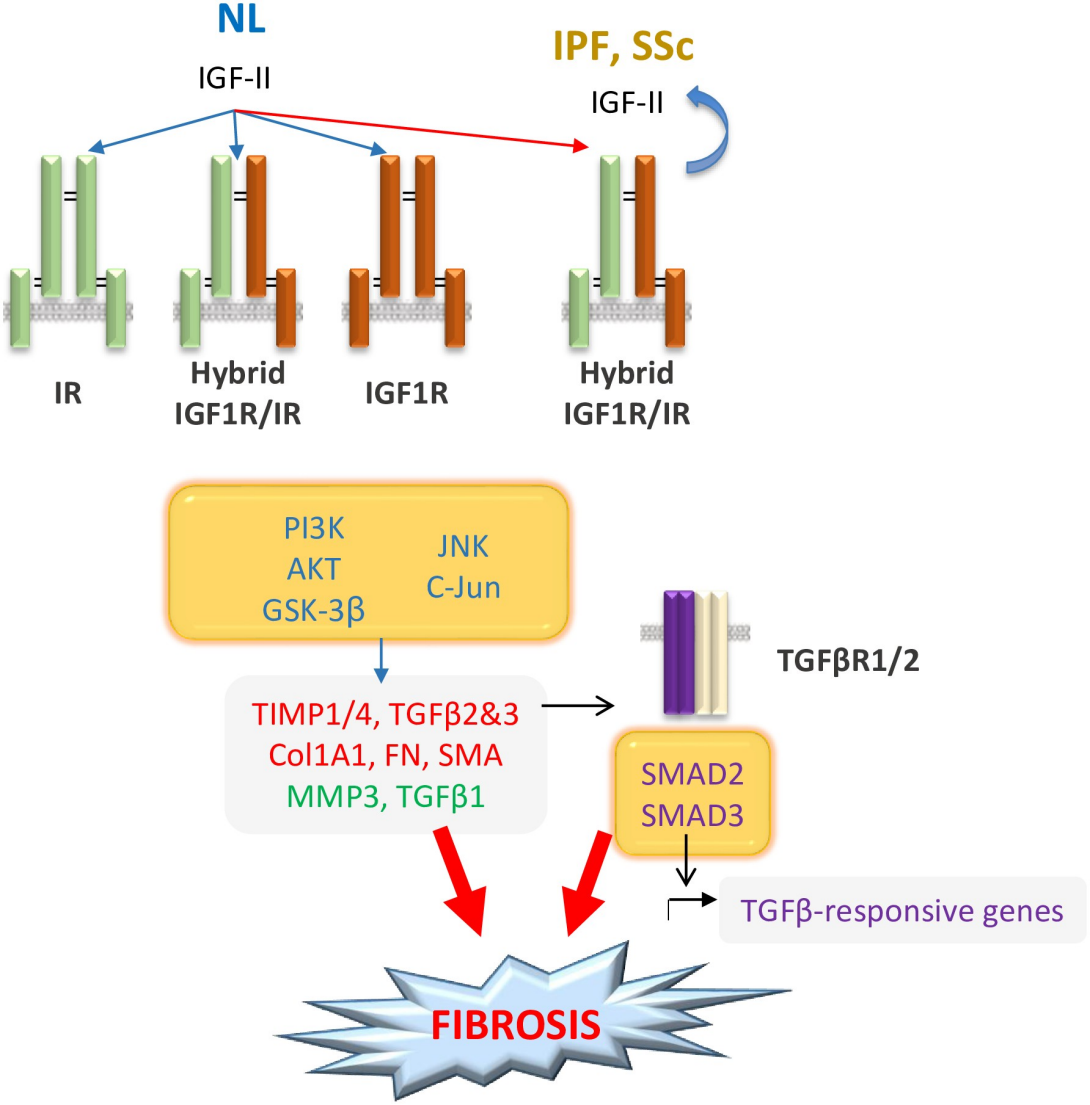
IGF-II mediates lung fibrosis through multiple mechanisms. IGF-II signals through IR, IGF1R, or hybrid receptors (IGF1R/IR), preferentially switching to the hybrid receptor species in IPF and SSc. Signaling via PI3K/AKT/GSK3β and JNK/c-Jun [12] results in upregulation of *ACTA2*, *TIMP1/4*, *Collagen*, *Fibronectin*, & *TGFβ2/3* and down-regulation of *MMP3* & *TGFβ1*. Stimulation by IGF-II also causes *TGFβ2/3* upregulation and canonical SMAD2/3 activation. Through differential gene expression and receptor species preferences, IGF-II induces formation of myofibroblasts and contributes to ECM accumulation, leading to fibrosis.

Having observed increased *TGFβ1* levels at baseline in IPF and SSc fibroblasts and induction of *TGFβ2* and *TGFβ3* levels by IGF-II, we examined the impact of inhibiting the shared TGFβ receptor on the IGF-II fibrotic response. In the presence of the TGFβ receptor inhibitor SB431542, the upregulation of αSMA, collagen, and fibronectin protein expression was significantly blocked, as was *ACTA2* and *collagen* gene expression. The positive control, TGFβ1, also led to significant increases in protein and mRNA that were abrogated with inhibitor. This additionally suggests that IGF-II mediates its fibrotic effects, at least in part, via TGFβ receptor activation. A schematic overview of our results is represented in Fig 7.

## Discussion

We recently showed that IGF-II levels were elevated in SSc and contribute to a pro-fibrotic condition through activation of PI3K and JNK signaling [12]. This afforded us the opportunity to study the role of IGF-II in mediating a pro-fibrotic signal through analysis of ligand-mediated changes in receptor expression, receptor activation, and production and secretion of components into the extracellular environment.

Receptor gene expression analyses under steady-state conditions revealed significantly decreased receptor levels in SSc compared to NL and IPF, with normal protein expression, yet SSc fibroblasts retained the ability to transduce the IGF-II signal. Significantly decreased receptor transcript levels in SSc lung fibroblasts with normal steady-state protein receptor levels could indicate high mRNA turnover, increased sensitivity to endogenous IGF-II expression, increased receptor protein half-life, altered nonsense-mediated decay, and/or decreased receptor degradation/turnover, thus affecting increased signal amplification downstream of the IGF-II: receptor interaction. With such a reduced pool of transcript available, SSc responses are likely rendered very sensitive to cognate ligand stimulation, possibly through signal amplification starting from the point of receptor transcription. This postulation aligns with our results of exogenous IGF-II- mediated stimulation inducing collagen and fibronectin transcription to a greater extent by SSc than by NL or IPF. Lack of differences in receptor protein expression between NL, IPF, and SSc suggests that dysregulation of receptor protein expression is itself not an underlying causative mechanism of fibrosis in IPF and SSc. Differential downregulation of IGF1R and IGF2R receptors by IGF-II stimulation may also indicate primary signaling through different receptor species in these populations of fibroblasts and highlights the differences between potential signal regulation and potential therapeutic targets in these two manifestations of fibrosing lung diseases. Several studies have shown that targeted disruption, deregulation, or altered imprinting of the *Igf2r* gene leads to increased IGF-II levels [31–33]; thus, the downregulated transcript levels of IGF-II receptors in SSc could be responsible for the increased systemic IGF-II historically observed in SSc patients. Additionally, others have shown that disruption of *Igf2r* or hypomethylation of the IGF-II P3 promoter (responsible for IGF-II-fibrotic signaling and whose mRNA is post-transcriptionally regulated [34]), result in higher levels of IGF-II secretion, rendering a sustained higher presence of growth factor in the environment, and ultimately leading to IGF-II-responsive receptors producing a variety of downstream effects [12, 32, 35–39]. Overall levels of IGF2R in SSc may be lower via two compounding mechanisms: reduced steady-state levels of *Igf2r* transcript, as well as the presence of systemic circulating autoantibodies capable of recognizing and targeting the IGF2R in SSc patients [40].

Functionally, we sought to determine the receptor(s) responsible for transducing the IGF-II signal leading to fibrosis through the use of neutralizing antibody, siRNA, and a targeted pharmacological inhibitor. In SSc fibroblasts, there is a less drastic change in collagen and fibronectin expression with IGF1R neutralization, knockdown, and inhibition compared to NL fibroblasts. Knockdown of IGF1R increased IR protein expression and vice versa in SSc, suggesting that an inverse compensation mechanism for the promotion of IGF-II-driven fibrosis exists in SSc fibroblasts. Based on these experiments, the primary species responsible for transducing the fibrotic signal leading to ECM secretion in SSc appears to be a combination of the IGF1R and IR receptors, possibly existing as an IGF1R/IR hybrid heteroreceptor form; whereas, there appears to be a greater contribution of IGF1R homodimers in addition to the possible IGF1R/IR hybrid receptors that transduce the IGF-II signal in NL, suggesting an IGF-II-driven receptor species shift in pathologic lung conditions associated with increased levels of IGF-II. This conclusion is supported by our finding of IGF-II-driven decrease in NL IR expression over time; this decrease in IR was absent in SSc, suggesting the accessible pool of IR receptors is maintained in the presence of IGF-II and available for the formation of hybrid receptors. The IGF1R/IR hybrid heterodimer has been reported to function more similarly to an IGF1R homodimer than an IR homodimer, with heterodimers assembled as efficiently as homodimers [15, 18, 20, 41, 42]. Both the IGF1R and the IR contain two potential binding pockets that exhibit negative cooperativity which may or may not be influenced by the dimerization state of the receptor (whether homodimerized or heterodimerized) [17]. Single nucleotide mutations in the ligand binding site of IGF1R have been associated with development of various cancers, including non-small-cell lung cancer and multiple myeloma [43, 44] and may merit exploration for a possible role in the development of fibrosing lung disease. Attenuation of bleomycin-induced lung injury was shown with IGF1R blockade and in an IGF1R-deficient mouse model, touting the important contribution of this receptor in IGF-II-mediated fibrosis [45, 46].

Herein we found a shift in the fibrotic ratio in IPF and SSc that favored the accumulation of ECM. The ECM is a complex milieu of growth factors, cytokines, macromolecules, fibrous proteins (including collagen and fibronectin), degradation enzymes (including MMPs) and their inhibitors (TIMPs) [47]. The presence, absence, or ratio of these components can direct changes in the cells in contact with the ECM, influencing cellular contouring, proliferation, migration/invasion, intracellular signal transduction, and survival. Supernatant transfer and conditioned media experiments have shown that the ECM environment can modulate the activities of the cells it surrounds [48, 49]. Through proteolytic cleavage, MMPs affect a variety of ECM proteins: activating zymogens, revealing obscured binding sites, and releasing bound growth factors, such as IGF-II and TGFβ [50–52]. MMP3 or stromelysin-1 is part of a family of proteases with an active site containing zinc and a conserved methionine residue [50, 53]. Fibroblast and murine studies have revealed that MMP3 plays a distinct role in wound healing, as fibroblasts exhibit defective contraction *in vitro* with slower incisional and full-thickness wound healing in MMP3-deficient mice compared to WT mice [54, 55]. TIMPs are a family of endogenous inhibitors of MMPs that have specific roles in modulating ECM proteolysis and turnover [56]. TIMP1 has been found to be increased transcriptionally and translationally in a mouse model of bleomycin-induced lung fibrosis [57]. While TIMP1 and TIMP4 are structurally similar and are both broadly inhibitive to MMPs, TIMP1 exhibits stronger affinity for MMP3 and can inhibit a greater number of MMPs [58, 59]. TIMP1 and TIMP4 were both found to correlate inversely with suppressor of cytokine signaling 1 (SOCS1) expression in two *in vitro* fibrosis model systems [60] and putatively shown to share CD63, an exosomal marker, as a binding partner in MMP-independent activities [61, 62]. Therefore, IGF-II may be repressing the expression of the TIMP repressor in addition to *MMP3* transcripts, while facilitating the type and scale of extracellular delivery of pro-fibrotic factors (preferentially switching from the more specific inhibitor TIMP4 in NL to the broader inhibitor TIMP1 in IPF and SSc, based on the sheer magnitude of secretion in IPF and SSc compared to NL, Fig 4). Interestingly, a case has been described in a child wherein the TIMP1:MMP3 ratio was tipped in the opposite direction [63]. Dermal fibroblasts isolated from the patient exhibited increased fibroblast proliferation and defective ECM deposition, though the authors did not scrutinize the potential contribution of IGF-II or its receptors. Knockdown of IGF-II in two inducible colorectal cancer cell lines decreased cellular adhesion to ECM proteins and downregulated several genes involved in cell-cell and cell-matrix contact [64], further emphasizing the role of IGF-II in ECM regulation; the effect of exogenous IGF-II addition to fibroblasts on adhesion molecules was not explored in this study, but could play a role in facilitating the establishment of anchoring proteins for ECM deposition and accumulation.

IGF-II stimulation caused upregulation of collagen and fibronectin earlier and to a greater extent in IPF and SSc compared to NL, suggesting that IPF and especially SSc fibroblasts are transcriptionally more sensitive to IGF-II stimulation. The upregulation of *collagen* and *fibronectin* mRNA directly correlated with the accumulation of these proteins in the extracellular milieu seen at 24–48 hr [12, 65]. Whereas Grotendorst *et al*. found that low levels of IGF-II (10 ng/mL) could produce fibrotic effects only in the presence of the quintessential fibrotic mediator TGFβ1, addition of exogenous TGFβ1 was not required for IGF-II to promote a pro-fibrotic condition and accumulation of ECM proteins in our current or previous study using primary human adult lung fibroblasts [66]. *TGFβ1* transcript levels were found to be elevated in unstimulated IPF and SSc lung fibroblasts compared to NL fibroblasts, and addition of IGF-II led to a time-dependent decrease in *TGFβ1* mRNA in NL, implying that IGF-II does not rely on induction of *TGFβ1* transcript or require supplemental addition of TGFβ to mediate fibrosis. As IPF and SSc fibroblasts have high levels of active/steady-state *TGFβ1* expression, our findings suggest that *in vivo* IGF-II is likely inducing *TGFβ2* and *TGFβ3*, thus contributing to and perpetuating the fibrotic phenotype.

While many *in vitro* experiments rely on TGFβ1 to promote fibrosis, TGFβ2 has been shown to mediate cardiac fibroblast differentiation into myofibroblasts [67] and to be epigenetically modified in SSc [68], while TGFβ3 has been reported to increase collagen I deposition and αSMA expression in dermal fibroblasts [69]. A previous report has additionally found an association between TGFβ2, TGFβ3, and TIMP1 in systemic sclerosis [70], which may ultimately be due to the contribution of IGF-II, based on the results of this study. This is the first report of IGF-II actively upregulating *TGFβ2* and *TGFβ3* isoforms and promoting a fibroblast-to-myofibroblast transition, implicating a role for IGF-II in wide-ranging biological processes involving myofibroblast formation and representing new experimental targets in pulmonary fibroses.

## Conclusion

Herein we have described IGF-II-stimulated changes in IGF-II receptors and intracellular and secreted ECM components in fibroblasts from normal lung, IPF, and SSc. Furthermore, we have proposed several mechanisms for IGF-II-mediated fibrosis, including shifting transducing receptor pairing, upregulating multiple TGFβ isoforms, signaling through SMAD2/3, downregulating antifibrotic mediators, and stimulating ECM deposition via a shift in TIMP species, which altogether alter the cellular milieu and promote a pro-fibrotic state.

## Supporting information

S1 Fig Donor ages, IGF-II stimulated ECM gene expression, and baseline active TGFβ1 levels. **A**: Age demographics of donor populations. Data was available from 70% of NL, 80% of IPF, and 90% of SSc donors. **B**: IGF-II (200 ng/mL)-stimulated gene expression of *Fibronectin* and *Collagen* at 24 hr and 48 hr in NL, IPF, and SSc fibroblasts. **C**: IGF-II increased levels of activated TGFB1 in 48 hr supernatants from NL, IPF, and SSc via ELISA. N = 5–10. *p<0.05, **p<0.01, ***p<0.001 compared to respective vehicle by 1-way ANOVA with Dunnett’s multiple comparison post-hoc test.
(TIF)

## Abbreviations

ECM: extracellular matrix
IGF1R: type 1 IGF receptor
IGF2R: type 2 IGF receptor
IGFBP: IGF binding proteins
IGF-II: Insulin-like growth factor II
IPF: idiopathic pulmonary fibrosis
IR: insulin receptor
MMP: matrix metalloproteinase
NL: normal lung
PBS: phosphate buffered saline
SSc: systemic sclerosis/scleroderma
TGFβ: transforming growth factor β
TIMP: tissue inhibitor of MMP
TKI: tyrosine kinase inhibitor
