# Supplementary figures and images for: Insulin-like growth factor (IGF)-II- mediated fibrosis in pathogenic lung conditions
Source: PLoS One. 2019 Nov 25;14(11):e0225422. doi: 10.1371/journal.pone.0225422 (PMC6876936; doi:10.1371/journal.pone.0225422)

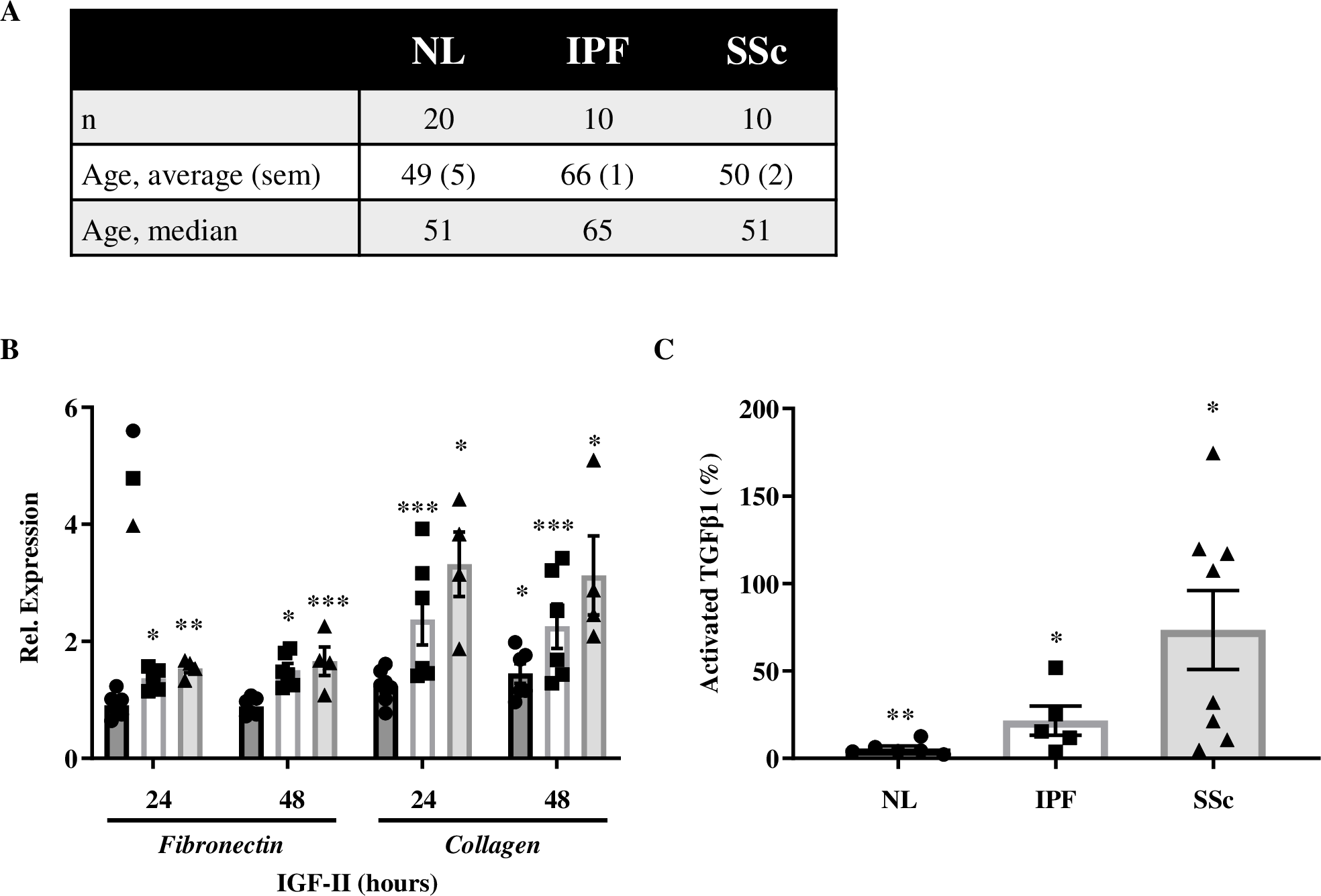

Supplement: S1 Fig — A: Age demographics of donor populations. Data was available from 70% of NL, 80% of IPF, and 90% of SSc donors. B: IGF-II (200 ng/mL)-stimulated gene expression of Fibronectin and Collagen at 24 hr and 48 hr in NL, IPF, and SSc fibroblasts. C: IGF-II increased levels of activated TGFB1 in 48 hr supernatants from NL, IPF, and SSc via ELISA. N = 5–10. *p<0.05, **p<0.01, ***p<0.001 compared to respective vehicle by 1-way ANOVA with Dunnett’s multiple comparison post-hoc test. (TIF) [file pone.0225422.s001.tif]
